# Monitoring the Bioprotective Potential of *Lactiplantibacillus pentosus* Culture on Pathogen Survival and the Shelf-Life of Fresh Ready-to-Eat Salads Stored under Modified Atmosphere Packaging

**DOI:** 10.3390/pathogens13070557

**Published:** 2024-07-02

**Authors:** Angeliki Doukaki, Olga S. Papadopoulou, Chrysavgi Tzavara, Aikaterini-Malevi Mantzara, Konstantina Michopoulou, Chrysoula Tassou, Panagiotis Skandamis, George-John Nychas, Nikos Chorianopoulos

**Affiliations:** 1 Laboratory of Microbiology and Biotechnology of Foods, Department of Food Science and Human Nutrition, School of Food and Nutritional Sciences, Agricultural University of Athens, Iera Odos 75, 11855 Athens, Greece; angieduk@gmail.com (A.D.); tzavarachr@gmail.com (C.T.); katerina.mantzara98@gmail.com (A.-M.M.); konstantinamich6@gmail.com (K.M.); gjn@aua.gr (G.-J.N.); 2 Institute of Technology of Agricultural Products, Hellenic Agricultural Organization–DIMITRA, S. Venizelou 1, Lycovrissi, 14123 Athens, Greece; olgapapadopoulou@elgo.gr (O.S.P.); ctassou@elgo.gr (C.T.); 3 Laboratory of Food Quality Control and Hygiene, Department of Food Science and Human Nutrition, School of Food and Nutritional Sciences, Agricultural University of Athens, Iera Odos 75, 11855 Athens, Greece; pskan@aua.gr

**Keywords:** pre-cut salads, biopreservation, *Listeria monocytogenes*, *Escherichia coli*, Fourier transform infrared (FTIR) spectroscopy, multispectral imaging (MSI) analysis

## Abstract

Globally, fresh vegetables or minimally processed salads have been implicated in several foodborne disease outbreaks. This work studied the effect of *Lactiplantibacillus pentosus* FMCC-B281 cells (F) and its supernatant (S) on spoilage and on the fate of *Listeria monocytogenes* and *Escherichia coli* O157:H7 on fresh-cut ready-to-eat (RTE) salads during storage. Also, Fourier transform infrared (FTIR) and multispectral imaging (MSI) analysis were used as rapid and non-destructive techniques to estimate the microbiological status of the samples. Fresh romaine lettuce, rocket cabbage, and white cabbage were used in the present study and were inoculated with *L. pentosus* and the two pathogens. The strains were grown at 37 °C for 24 h in MRS and BHI broths, respectively, and then were centrifuged to collect the supernatant and the pellet (cells). Cells (F, ~5 log CFU/g), the supernatant (S), and a control (C, broth) were used to spray the leaves of each fresh vegetable that had been previously contaminated (sprayed) with the pathogen (3 log CFU/g). Subsequently, the salads were packed under modified atmosphere packaging (10%CO_2_/10%O_2_/80%N_2_) and stored at 4 and 10 °C until spoilage. During storage, microbiological counts and pH were monitored in parallel with FTIR and MSI analyses. The results showed that during storage, the population of the pathogens increased for lettuce and rocket independent of the treatment. For cabbage, pathogen populations remained stable throughout storage. Regarding the spoilage microbiota, the *Pseudomonas* population was lower in the F samples, while no differences in the populations of *Enterobacteriaceae* and yeasts/molds were observed for the C, F, and S samples stored at 4 °C. According to sensory evaluation, the shelf-life was shorter for the control samples in contrast to the S and F samples, where their shelf-life was elongated by 1–2 days. Initial pH values were ca. 6.0 for the three leafy vegetables. An increase in the pH of ca. 0.5 values was recorded until the end of storage at both temperatures for all cases of leafy vegetables. FTIR and MSI analyses did not satisfactorily lead to the estimation of the microbiological quality of salads. In conclusion, the applied bioprotective strain (*L. pentosus*) can elongate the shelf-life of the RTE salads without an effect on pathogen growth.

## 1. Introduction

Nowadays, the daily consumption of fresh produce (i.e., lettuce, cabbage, etc.) has been associated with healthy eating in consumer lifestyles. However, consumers are demanding more convenient foods with minimum preparation time due to their busy lifestyles, so the food industry must meet the growing interest in ready-to-eat (RTE) fresh vegetables. A high concern for the food industry is the high hygienic standards of the products that are released onto the market. In that sense, the products must be free of pathogenic microorganisms and have the lowest possible load of microbial flora to avoid rapid spoilage. After harvest, the fresh leafy vegetables undergo simple interventions (i.e., cleaning, washing, cutting, and packing) to meet the hygienic criteria for consumption, and therefore, their high microbiological quality is of great concern [1,2]. Among the interventions that are taking place in preparing RTE fresh vegetables, the main factor that is responsible for fast deterioration during storage of the fresh produce is the cutting procedure. Thus, producers of leafy vegetables must follow good agricultural practices and implement hygienic measures during the cultivation process, such as quality control of the irrigation water, worker hygiene and quality control of organic fertilizers, otherwise there is an increased risk in contamination by pathogenic microorganisms.

Leafy vegetables, which are most often eaten raw, have been linked with various foodborne infections due to contamination with human enteric pathogens, such as *Escherichia coli* O157:H7 and *Listeria monocytogenes* [3]. A recent review [4] noted that Norovirus, Shiga toxin-producing *Escherichia coli* (both non-O157 and O157:H7), *Campylobacter* spp., and nontyphoidal *Salmonella* were associated with the highest number of illnesses after the consumption of leafy vegetables in the USA. In addition, except Shiga toxin-producing *Escherichia coli*, other pathogens such as *Salmonella*, *Shigella*, *Yersinia enterocolitica*, and *L. monocytogenes* have been implicated in outbreaks associated with leafy vegetables from 2000 onwards in Europe, the USA, and Canada, as reported in Mogren et al. [5]. As reported above, many steps, starting from the cultivation to the packaging of the fresh vegetables, can be responsible for contamination with foodborne pathogens [6,7,8,9], and since animal husbandry is usually practiced next to agricultural facilities, the animals are considered as the main source of pathogenic contamination, thus increasing the risk of product contamination [10]. Recently, several outbreaks of foodborne illness associated with leafy vegetables, especially lettuce, have been reported worldwide [11,12]. For instance, Yang and Scharff [4] estimated illness incidence and economic cost models for leafy vegetables in the USA and observed that lettuce takes 60.8% of leafy vegetables outbreaks, it accounts for up to 75.7% of leafy vegetables foodborne illnesses, and it accounts for 70% of the costs, while *Escherichia coli* O157:H7 (STEC) illnesses are related to romaine, which causes 12,496 approximate illnesses and costs $324.64 million annually in the USA. Finally, in a survey conducted at the Cypriot market, 216 RTE salads were analyzed, and the results showed that *E. coli* and *L. monocytogenes* were present in 11.57% and 3.70% of samples, respectively, whereas *Bacillus cereus* was found in 9.26% of samples [13], highlighting the need for the continuous investigation of their safety.

Today, due to the transportation of agricultural products over long distances (i.e., exports to other countries), the time from harvest to consumption also expands, resulting in an increase in their microbial load. Thus, the need for interventions to limit the growth of microbial populations throughout the distribution channel is necessary. Currently, microbial reduction interventions for leafy vegetables only involve antimicrobial washes. Usually, chlorine is used, but many studies have reported that chlorine has limited antimicrobial efficacy, mostly due to the low levels permitted. Furthermore, chlorine use can result in the production of potentially toxic compounds [14,15,16,17,18].

Increased consumer concern regarding the use of chemical disinfection methods in foods has led the food industry to search for mild alternative methods to enhance food safety and quality [19]. Biopreservation is such a method, mainly using protective lactic acid bacteria (LAB) cultures, which are generally recognized as safe (GRAS), to improve the quality and safety of fresh and minimally processed vegetables [16,17,18,20,21,22,23,24]. The preservation abilities of LAB can be attributed to the production of metabolites with known antimicrobial action, such as organic acids, hydrogen peroxide, and bacteriocins [25]. For instance, bacteriocins are considered safe biopreservatives and can inhibit the growth of other bacteria [26,27]. *L. pentosus* FMCC-B281 was used in the current study, and the selection of this strain was based on its in vitro probiotic properties [28,29] and its high survival rate in vegetable products stored in MAP [30].

As mentioned above, due to the transportation of agricultural products such as leafy vegetables over long distances, there is a high possibility of an increased microbial load at the time of consumption. It is of great concern for the food industry to control the initial microbial population at the very first steps of processing and, consequently, organize the products transportation and distribution according to their spoilage potential. In this context, the existing microbiological analyses, which are costly, require high-tech instruments and highly trained personnel, and provide results after 48 h, cannot cover the gap for immediate decisions. Also, those analyses are usually destructive to test products, limiting their potential to be used on-, in-, or at-line [31]. These disadvantages, in combination with the rapid evolution of technology, have led to the pursuit of new methodologies in the food industry. In that framework, spectroscopy-based sensors combined with machine learning methodologies and their ability to be used online have been investigated [31] and have shown that they may estimate very quickly the microbiological quality and, by extension, the shelf-life of various food products [32,33]. Nevertheless, sometimes they lack the ability to predict the shelf-life, depending on the food type.

The purpose of the present work was to study the effect of *Lactiplantibacillus pentosus* FMCC-B281 cells and their supernatant on spoilage and on the fate of *Listeria monocytogenes* and *Escherichia coli* 0157:H7 on fresh-cut salads (ready-to-eat) during storage using a holistic approach of analyses. In this respect, the feasibility of Fourier-transform infrared spectroscopy (FTIR) and multispectral imaging (MSI) to assess the microbiological quality of salads was also explored.

## 2. Materials and Methods

### 2.1. Experimental Design

Three (3) fresh-cut salads (white cabbage—WC, romaine lettuce—RL, and rocket salad—RS) were inoculated with two (2) foodborne pathogens, separately (cocktail strains of *Escherichia coli* O157:H7 or *Listeria monocytogenes*) and were also sprayed with *Lactiplantibacillus pentosus* FMCC-B281 cells or their supernatant, to study its effect as a bioprotective culture during storage. Samples were subjected to microbiological, pH, sensory, image, and spectroscopic analyses. 

### 2.2. Preparation of the Bacterial Cultures

In the current study, *Lactiplantibacillus pentosus* strain FMCC-B281, *Listeria monocytogenes strains* FMCC-B127 (designation 21075, chicken salad isolate), FMCC-B133 (designation 21736, soft cheese isolate), *Escherichia coli* O157:H7 strains FMCC-Β289 (ATCC-35150), and FMCC-Β290 (*Escherichia coli* K12xB DB3.1) were used (all strains belong to the culture collection of Agricultural University of Athens, Greece). In brief, monocultures of the selected strains were revived from stock cultures containing 20% glycerol that were stored at −80 °C. Each strain of the foodborne pathogens was inoculated in Tryptone Soy Broth (TSB, NCM0019A, Neogen, Kansas, MO, USA) and incubated overnight at 37 °C. A subculture of each strain was prepared in 10 mL of fresh TSB and incubated for 18 h at 37 °C. Biomass of the cells was then harvested using centrifugation (6000× rpm, 10 min, 4 °C), the pellet was washed twice with sterile ¼ strength Ringer’s solution (NCM0191K, Neogen), and the final pellet was resuspended in Ringer’s solution. The biomass of the strains of each pathogen was mixed in equal volumes, and serial dilutions were prepared to inoculate the fresh-cut salads with a population of approximately 3 log CFU/g. The inoculum size was confirmed before use with the serial dilution method, and the inoculants were spread on Harlequin^®^ Tryptone Bile Glucuronide Agar (TBX) (NCM1001, Neogen) and *Listeria* Palcam Agar (Ref. 4016042, Biolife, Italiana S.r.l, Milano, Italy) with Palcam selective supplement (*Listeria* Palcam Antimicrobic Supplement, Ref. 4240042, Biolife) for *E. coli* and *L. monocytogenes*, respectively.

For *L. pentosus*, the stock culture was inoculated in de Man, Rogosa, and Sharpe (MRS) broth (MRS broth, 4017292, Biolife, Milano, Italy) and incubated overnight at 30 °C. A subculture was prepared in sterile 10 mL vegetable extract broth (as described in Section 2.3) and incubated for 24 h at 30 °C. The cells were then harvested using centrifugation (6000× rpm, 10 min, 4 °C), and the supernatant was collected under aseptic conditions to be further used for inoculation of the salads. The collected supernatant was sterilized through a 0.22 filter (Millipore PVDF Syringe filter/L0.22 μm). The pellet of the microorganism was also collected and subsequently washed with Ringer, as described above. The pellet was then diluted in the appropriate amount of vegetable sterile extract broth to inoculate the fresh-cut salads with a population level of 5 log CFU/g. Inoculum size was confirmed before use with the serial dilution method after pour plating of the inoculants on MRS ISO agar (NCM0190, Neogen).

### 2.3. Inoculation of Fresh-CUT Salads and Packaging of the Samples

Fresh white cabbage (*Brassica oleracea*), romaine lettuce (*Lactuca sativa* L. var. *longifolia*), and rocket salad (*Eruca sativa* L.) were purchased from a local public market (Athens, Greece) and transported to the laboratory within 30 min to be used on the same day. First, the outer (4–6) leaves, or injured leaves, of the salads were removed, and accordingly, tap water was used for washing to remove any organic material. Then, the leaves were dried inside a laminar flow cabinet for approximately half an hour. Accordingly, the salads were cut into strips (approximately 1–3 cm in width, depending on the vegetable) inside the laminar flow cabinet using sterile kitchen scissors and were divided into portions of 30 g.

A vegetable sterile extract broth was prepared exactly as described by Manios et al. [34]. This sterile broth (containing leaf extracts from WC and RL) was used as (i) the broth to spray the leaves of the control samples and (ii) the medium to grow the subculture of *L. pentosus* that was further used to spray the leaves (both cells and supernatant).

Inoculation of the salads either with *L. monocytogenes* strains or with *E. coli* strains followed by spraying the pathogen (300 μL of a 5 log CFU/mL culture of the pathogen to spray 30 g of cut leaves) on the fresh-cut leaves was conducted to result in a population of 3 log CFU/g in the final product. Then, the salads were sprayed with (i) the vegetable sterile extract broth by spraying 300 μL to 30 g of fresh-cut leaves (to serve as the control), (ii) the supernatant of *L. pentosus* by spraying 300 μL supernatant to 30 g of fresh-cut leaves (that were acquired after an overnight incubation of the strain in the vegetable sterile extract broth and were separated from the pellet with centrifugation, see Section 2.2), and (iii) the cells of *L. pentosus* that have been diluted in the vegetable sterile extract broth using 300 μL of a 7 log CFU/mL culture of *L. pentosus* to spray 30 g of cut leaves and were thoroughly mixed for the equal dispersion of the strains. Then, the salads were left for 5–10 min to absorb the cultures or the supernatants and packaged in sterile pouches (in portions of 30 g each) under modified atmosphere packaging (MAP, 10% CO_2_/10%O_2_/80% N_2_) using a HenkoVac 1900 machine (Howden Food Equipment B.V., the Netherlands). Samples were then stored at 4 °C and at 10 °C in high precision incubators (MIR153, Sanyo Electric Co., Osaka, Japan) until spoilage.

### 2.4. Microbiological Analysis and pH Measurements

Microbial analyses of the fresh-cut salads were carried out from day 0 to day 12, depending on the sample and storage temperature, according to Manios et al. [34]. In brief, 10 g of duplicate samples were aseptically added to 90 mL of sterilized ¼ Ringer’s solution in sterile stomacher bags (BagLight^®^, INTERSCIENCE, Paris, France) and homogenized using a Stomacher (Lab Blender 400, Seward Medical, London, UK) for 60 sec at room temperature. Then, appropriate serial dilutions were prepared using the same diluent to monitor the growth of the inoculated bacteria and the indigenous microbiota. Accordingly, 0.1 or 1 mL of inoculants were spread or poured on the following non-selective and selective agar media: Plate Count Agar (Tryptic Glucose Yeast Agar PCA, Ref. 4021452, Biolife, Italiana S.r.l, Milano, Italy) for the enumeration of Total Aerobic Viable Counts (TVC), incubated at 30 °C for 48–72 h; MRS ISO Agar (Neogen) overlaid with the same medium and incubated at 30 °C for 48–72 h for the enumeration of mesophilic lactic acid bacteria (LAB); *Pseudomonas* Agar Base (LAB108, LAB M., Burry, U.K.) with selective supplement Cetrimide-Fusidin-Cephaloridine (CFC) (Modified C.F.C X108, LAB M) for the enumeration of *Pseudomonas* spp., incubated at 25 °C for 48–72 h; Rose Bengal Chloramphenicol agar (RBC; LAB036, LAB M, Lancashire, UK) for the enumeration of yeasts/molds, supplemented with selective supplement X009 (LAB M), incubated at 25 °C for 48–72 h; Violet Red Bile Glucose Agar (VRBGA, Ref. 4021862, Biolife) for the enumeration of *Enterobacteriaceae* counts overlaid with the same medium and incubated at 37 °C for 18–24 h; Harlequin^®^ Tryptone Bile Glucuronide Agar (TBX) (Neogen) for the enumeration of *E. coli*, incubated at 44 °C for 18–24 h; and *Listeria* Palcam Agar Base (Biolife) with Palcam selective supplement (Biolife) for the enumeration of *L. monocytogenes* incubated at 37 °C for 24 and 48 h. Control (not inoculated) samples were also checked for the absence of the target pathogens.

After the microbiological analyses, the pH value of the fresh-cut salads was recorded with a digital pH meter Russell RL150 (Russell Inc., Cork, Ireland), and the glass electrode (Metrohm AG, Herisau, Switzerland) was immersed in the homogenized sample (stomacher bag, 1st decimal dilution).

### 2.5. Sensory Analysis

Sensory analysis was conducted by a semi-trained panel of nine people, representing the preferences of the average consumer. All panelists provided consent prior to their participation in the study. Features that were evaluated were the appearance (color and rigidity of the leaves) and the smell (familiar smell of the vegetables, rancid smell of the samples, and/or musty smell of the samples). A three-class evaluation scheme was used in this experiment, in which the first class (0–1, fresh) corresponded to fresh samples; the second class (1.5–2, marginal) corresponded to semi-fresh samples (2~accepted); and the third class (2.5–3, unacceptable) corresponded to spoiled samples (the three-class evaluation scheme used was based on previous works of our laboratory, i.e., Papadopoulou et al. [35]). The samples that were negatively evaluated (by at least half of the sensory panel) in one feature were considered unacceptable, and this point was considered the end of the shelf-life of the sample.

### 2.6. FTIR-ATR Spectroscopy

FTIR analysis was conducted using a ZnSe 45° HATR (Horizontal Attenuated Total Reflectance) crystal (PIKE Technologies, Madison, WI, USA) and an FTIR-6200 JASCO spectrometer (Jasco Corp., Tokyo, Japan) equipped with a standard sample chamber, a triglycine sulfate (TGS) detector, and a Ge/KBr beamsplitter. A sufficient volume of sample was placed on the crystal plate to cover it completely. The crystal used had a refractive index of 2.4 and a depth of penetration of 2.0 μm at 1000 cm^−1^. Using the Spectra Manager™ Code of Federal Regulations (CFR) software version 2 (Jasco Corp.), spectra were collected over the wavenumber range of 4000 to 400 cm^−1^ by accumulating 100 scans with a resolution of 4 cm^−1^ and a total integration time of 2 min. Prior to the measurements of the tested samples, reference spectra were acquired using the cleaned blank crystal (i.e., no added sample). After each measurement, the crystal’s surface was cleaned, first with detergent and distilled water and then with analytical grade acetone, and dried using lint-free tissue. The FTIR spectra used for further analysis ranged between 1800 and 870 cm^−1^. In total, 263 samples were acquired for cabbage, 190 for lettuce, and 220 for rocket. 

### 2.7. Image Acquisition and Segmentation

Multispectral image (MSI) analysis was carried out using the VideometerLab and VideometerLite instruments (Videometer A/S, Herlev, Denmark). The VideometerLab instrument collects spectral data in 18 different wavelengths (405–970 nm) from the acquired image. A more detailed description of the organology and calibration of the VideometerLab instrument can be found elsewhere [36]. The VideometerLite is a portable and wireless instrument that acquires images in seven different wavelengths ranging from 405 nm to 850 nm. Analytically, these wavelengths are 405, 460, 535, 590, 621, 660, and 850 nm.

**Figure 1:**
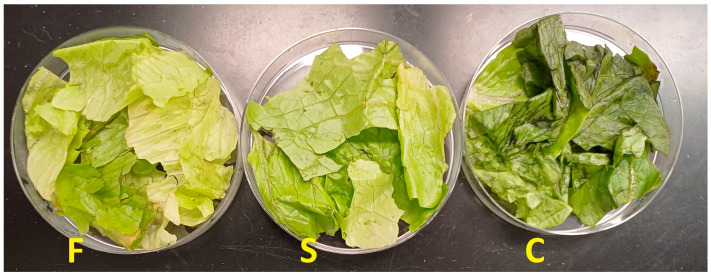
Images of cells (F), supernatant (S), and control (C) lettuce samples after 6 days of storage at 4 °C.

The samples were placed into Petri dishes (Figure 1) and then transferred inside the Ulbricht sphere of the VideometerLab or manually placed under the sphere of the portable VideometerLite, both of which have a top-mounted camera, and then, multispectral images of the samples were taken. The VideometerLab system software (version 2.12.39) was used for the selection of the region of interest (ROI) of the samples from non-relevant areas (e.g., sample background, Petri dish). Canonical discriminant analysis (CDA) was employed, resulting in segmented images. The outcome of this process was the calculated average value and standard deviation of the intensity of the pixels within the ROI at each wavelength. From VideometerLab, 325 samples in total were acquired for cabbage, 230 for rocket, and 267 for lettuce. From VideometerLite, 157 samples were acquired for cabbage, 119 for rocket, and 130 for lettuce.

### 2.8. Data Analysis

Partial least squares regression—PLS-R—was used on spectroscopic data for the prediction of total viable counts. Stratified sampling was used on the data, where 70% of each dataset was used for training the models and 30% for prediction. The PLS regression model was evaluated by the number of latent components that were extracted from the data, and this was determined using a leave-one-out cross validation procedure on the training set only. The number of latent components needed to yield the lowest root mean square error (RMSE) of the cross-validation was evaluated for the modeling, examining in parallel the plot of cross-validation residual variance against the number of latent components, with up to 20 components included. In the case that the residual variance no longer decreased with additional components, the number of latent components of the first minimum value of residual variance was selected to avoid overfitting.

### 2.9. Evaluation of Model Performance

PLS-R was used, and its performance was compared based on the root mean square error (RMSE), the coefficient of determination (R^2^) for the actual values versus the prediction of the TVC counts. Furthermore, the bias (B_f_) and accuracy (A_f_) factors were utilized to assess the microbial counts [28,35].

### 2.10. Statistical Analysis

The experiments on fresh-cut salads were conducted using two independent batches with two replicates in each batch. Data were transformed to log CFU/g, and the average and standard deviation (SD) of log CFU/g were calculated for each sample point. For data analytics, the software XLSTAT^®^ software (version 2023.3.1, Addinsoft, New York, NY, USA) was used.

## 3. Results

### 3.1. Microbial Analyses and pH

**Figure 2:**
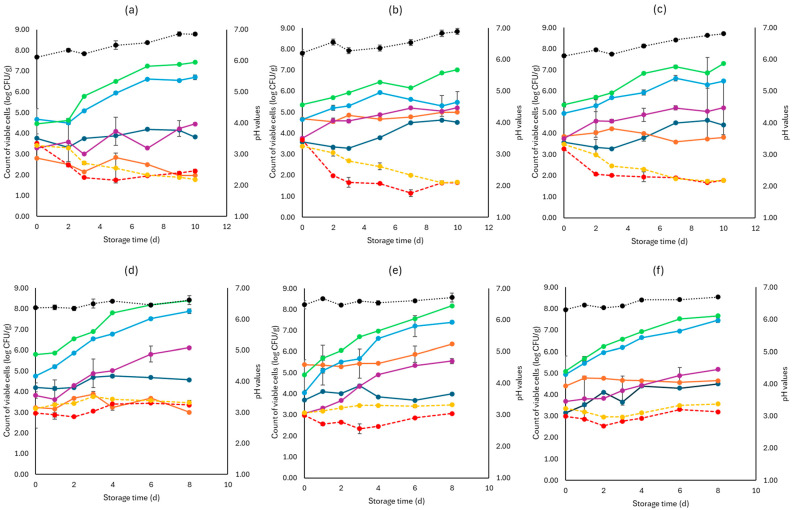
The population of the examined microorganisms and pH values in white cabbage samples (mean values ± standard deviations) for (**a**): C samples stored at 4 °C, (**b**): F samples stored at 4 °C, (**c**): S samples stored at 4 °C, (**d**): C samples stored at 10 °C, (**e**): F samples stored at 10 °C, and (**f**): S samples stored at 10 °C. (**•**) Total viable counts, (**•**) *Pseudomonas* spp., (**•**) *Enterobacteriaceae*, (**•**) Lactic acid bacteria, (**•**) Yeasts and molds are represented by a continuous line (-). *Listeria monocytogenes* (**•**) and *E. coli* 0157:H7 (**•**) are represented in dashed lines (---). The pH values (**•**) are indicated in the secondary axis and are represented with a dotted line (…).

**Figure 3:**
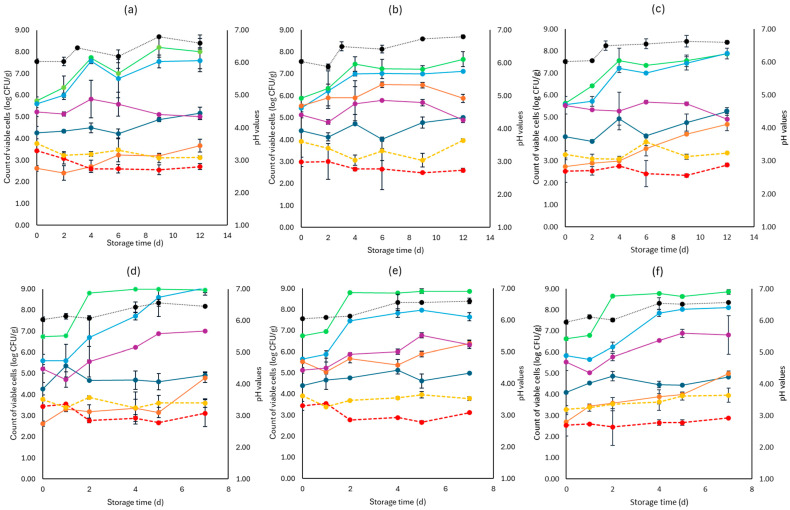
The population of the examined microorganisms and pH values in romaine lettuce samples (mean values ± standard deviations) for (**a**): C samples stored at 4 °C, (**b**): F samples stored at 4 °C, (**c**): S samples stored at 4 °C, (**d**): C samples stored at 10 °C, (**e**): F samples stored at 10 °C, and (**f**): S samples stored at 10 °C. (**•**) Total viable counts, (**•**) *Pseudomonas* spp., (**•**) *Enterobacteriaceae*, (**•**) Lactic acid bacteria, (**•**) Yeasts and molds are represented by a continuous line (-). *Listeria monocytogenes* (**•**) and *E. coli* 0157:H7 (**•**) are represented in dashed lines (---). The pH values (**•**) are indicated in the secondary axis and are represented with a dotted line (…).

**Figure 4:**
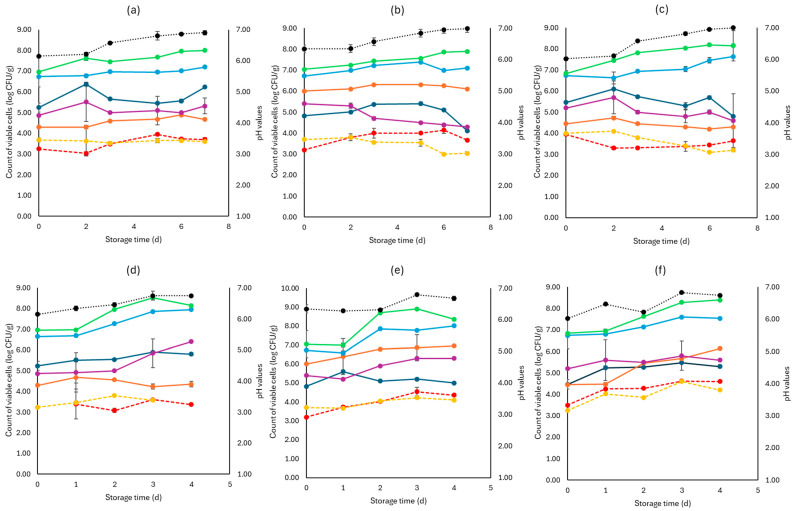
The population of the examined microorganisms and pH values in rocket samples (mean values ± standard deviations) for (**a**): C samples stored at 4 °C, (**b**): F samples stored at 4 °C, (**c**): S samples stored at 4 °C, (**d**): C samples stored at 10 °C, (**e**): F samples stored at 10 °C, and (**f**): S samples stored at 10 °C. (**•**) Total viable counts, (**•**) *Pseudomonas* spp., (**•**) *Enterobacteriaceae*, (**•**) Lactic acid bacteria, (**•**) Yeasts and molds are represented by a continuous line (-). *Listeria monocytogenes* (**•**) and *E. coli* 0157:H7 (**•**) are represented in dashed lines (---). The pH values (**•**) are indicated in the secondary axis and are represented with a dotted line (…).

Figure 2, Figure 3 and Figure 4 display the population of the examined microorganisms and the pH values for the cabbage, lettuce, and rocket samples. For cabbage, at day 0, the TVC was 5.08 log CFU/g (±0.42). As seen in Figure 2, the main spoilage microorganisms for both storage temperatures (4 and 10 °C) were *Pseudomonas* spp. and *Enterobacteriaceae*. The initial population of *Pseudomonas* spp. was 4.78 log CFU/g (±0.06) and for *Enterobacteriaceae* 3.44 log CFU/g (±0.15). For F samples at the end of storage at 4 and 10 °C, *Pseudomonas* spp. reached high population levels (6.67 ± 0.03 log CFU/g, 7.39 ± 0.07 log CFU/g, respectively), followed by *Enterobacteriaceae* (5.03 ± 1.28 log CFU/g, 5.55 ± 0.15 log CFU/g, respectively).

At the end of storage (4 and 10 °C) for the S samples, the population levels of *Pseudomonas* spp. (6.47 ± 0.5 log CFU/g, 7.46 ± 0.12 log CFU/g, respectively) as well as *Enterobacteriaceae* (5.21 ± 0.01 log CFU/g, 5.18 ± 1.25 log CFU/g, respectively) were close to those of the F samples. C sample populations for the end of storage (4 and 10 °C), for *Pseudomonas* spp. (6.70 ± 0.11 log CFU/g, 7.89 ± 0.06 log CFU/g, respectively) and *Enterobacteriaceae* (4.45 ± 0.05 log CFU/g, 6.12 ± 0.21 log CFU/g, respectively), did not show differences compared to the F and S samples. Yeasts and molds had an initial population of 3.68 log CFU/g (±0.21) and showed relatively stable growth at both storage temperatures for all samples (F, S, and C), as presented in Figure 2. The LAB population at the beginning of storage was higher in the F (5.03 ± 0.04 log CFU/g) and S samples (4.28 ± 0.17 log CFU/g) compared to the C samples (3.02 ± 0.30 log CFU/g), as expected. At the end of storage for both temperatures (4 and 10 °C), the F samples showed higher population counts (5.92 ± 0.05 log CFU/g, 5.35 ± 0.04 log CFU/g, respectively), while for the S samples (3.81 ± 0.23 log CFU/g, 4.64 ± 0.07 log CFU/g, respectively) and C samples (1.98 ± 0.03 log CFU/g, 3.00 ± 0.23 log CFU/g, respectively), the LAB population decreased. The growth of both pathogens examined was relatively stable.

More specifically, at 4 °C, *L. monocytogenes* showed a slightly lower population at the end of storage for the F (1.65 ± 0.05 log CFU/g) and S (1.78 ± 0.01 log CFU/g) samples compared to the C samples (2.18 ± 0.08 log CFU/g). At 10 °C, no significant difference was observed at the end of storage for the F (3.06 ± 0.02 log CFU/g), S (3.20 ± 0.15 log CFU/g) and C (3.65 ± 0.16 log CFU/g) samples. For *E. coli* 0157:H7, at the end of storage for both temperatures (4 and 10 °C), the population values of the F (1.68 ± 0.05 log CFU/g, 3.48 ± 0.16 log CFU/g. respectively), S (1.77 ± 0.02 log CFU/g, 3.56 ± 0.04 log CFU/g, respectively), and C (1.78 ± 0.04 log CFU/g, 3.47 ± 0.08 log CFU/g, respectively) samples were similar. The pH values were similar at both storage temperatures for all B, F, and C samples.

For lettuce, the TVC population at the beginning of storage was 6.46 log CFU/g (±0.36). Like cabbage, the main spoilage microorganisms for both storage temperatures (4 and 10 °C) were *Pseudomonas* spp. and *Enterobacteriaceae*, as depicted in Figure 3. At the end of storage at 4 °C, *Pseudomonas* spp. and *Enterobacteriaceae* had similar values for the F (7.11 ± 0.03 log CFU/g, 4.88 ± 0.11 log CFU/g, respectively), S (7.89 ± 0.24 log CFU/g, 4.89 ± 0.25 log CFU/g, respectively), and C (7.69 ± 0.52 log CFU/g, 5.00 ± 0.13 log CFU/g, respectively) samples. In a similar manner, at the end of storage at 10 °C, no differences were observed for the F (7.65 ± 0.20 log CFU/g, 6.33 ± 0.18 log CFU/g, respectively), S (8.12 ± 0.04 log CFU/g, 6.82 ± 0.91 log CFU/g, respectively), and C (9.00 ± 0.34 log CFU/g, 7.01± 0.04 log CFU/g, respectively) samples.

Yeasts and molds had an initial population of 4.26 log CFU/g (±0.05) and showed stable growth for all cases examined. As expected, the initial LAB population was the highest in the F (5.53 ± 0.01 log CFU/g) samples compared to the S (2.72 ± 0.05 log CFU/g) and C (2.61 ± 0.04 log CFU/g) and followed a relatively stable growth across all cases. As noticed in Figure 3, *L. monocytogenes* and *E. coli* 0157:H7 growth at the two storage temperatures (4 and 10 °C) did not differ significantly between the F, S, and C samples, while pH values were ca. 0.5 values higher from the initial pH (6.0) until the end of storage. The rocket samples had an initial TVC population of 6.98 log CFU/g (±0.23).

As seen in Figure 4, *Pseudomonas* spp. was the main spoilage microorganism at both temperatures (4, 10 °C), and its population at the end of storage for the F (7.15 ± 0.02 log CFU/g, 8.01 ± 0.18 log CFU/g), S (7.63 ± 0.04 log CFU/g, 7.54 ± 0.11 log CFU/g), and C (8.01 ± 0.41 log CFU/g, 7.95± 0.14 log CFU/g) samples was similar. The *Enterobacteriaceae* and yeasts/molds had close population values across storage at both temperatures. Pathogen populations (*L. monocytogenes* and *E. coli* 0157:H7) had no significant differences between the F, S, and C values for both temperatures.

### 3.2. Sensory Evaluation

**Figure 5:**
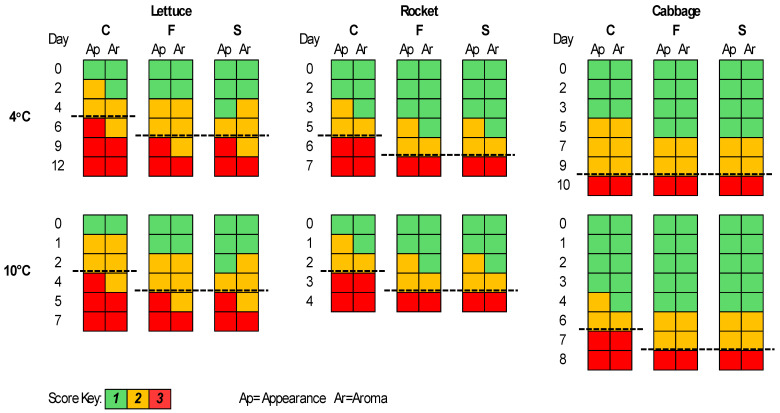
The sensory scores for romaine lettuce, rocket, and white cabbage samples during storage at 4 and 10 °C. Dashed lines represent the end of shelf-life.

The sensory evaluation results are presented in Figure 5. For lettuce, the F and S samples were better compared to the C samples regarding appearance and aroma for both storage temperatures. More specifically, the shelf-life of the F and S samples was elongated by 1–2 days, while the S samples had a better score than the F samples for appearance at some time points. The shelf-life of each case was estimated based on the time point at which the corresponding sample was organoleptically rejected. Thus, rocket seemed to have better score values for the F and S samples compared to the C for both appearance and aroma parameters and an elongation of its shelf-life of 1 day at both temperatures. For the cabbage samples at 4 °C, the F, S, and C samples were rejected at the same time point; however, at day 5, the F and S samples had lower scores for appearance and aroma in contrast to the C samples. At 10 °C, the F and S samples had an elongated shelf-life of 1 day.

### 3.3. Prediction of Microbial Quality

The total viable counts (TVC) in rocket, white cabbage, and romaine lettuce samples were predicted using partial least squares regression (PLS-R). Table 1 displays the performance indices of the models for training, validation, and prediction. The RMSE and R^2^ values of all datasets and cases examined for the prediction ranged from 0.555 to 1.464 and 0.041 to 0.756, respectively. The portable-MSI showed the best performance of all the instruments for prediction in all the cases examined: romaine lettuce (RMSE = 0.604, R^2^ = 0.347), white cabbage (RMSE = 0.722, R^2^ = 0.756), and rocket (RMSE = 0.555, R^2^ = 0.387). The FTIR at the examined wavenumbers (1800–870 cm^−1^) and the benchtop-MSI showed moderate performance compared to the portable-MSI, with ranges of RMSE 0.572 to 1.464 and R^2^ 0.041 to 0.331 for the prediction. Overall, the PLSR models were not satisfactory for the estimation of TVC counts.

**Figure 6:**
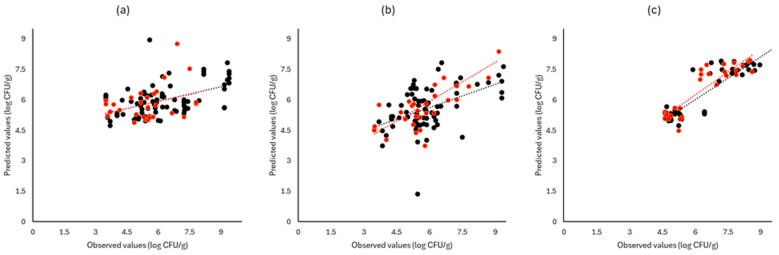
Observed versus predicted values for the estimation of TVC counts on white cabbage samples for FTIR (**a**), benchtop-MSI (**b**), and portable-MSI (**c**). Prediction (•), Validation (•). Dashed lines represent the linear trendline.

**Figure 7:**
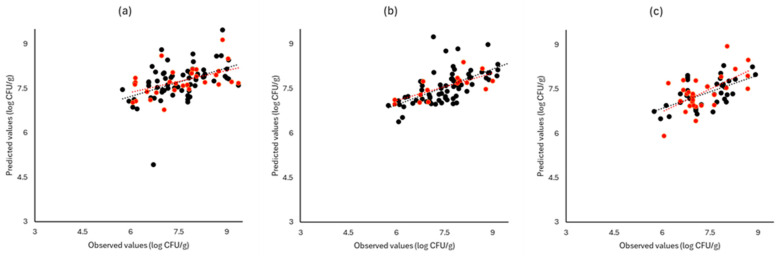
Observed versus predicted values for the estimation of TVC counts on romaine lettuce samples for FTIR (**a**), benchtop-MSI (**b**), and portable-MSI (**c**). Prediction (•), Validation (•). Dashed lines represent the linear trendline.

**Figure 8:**
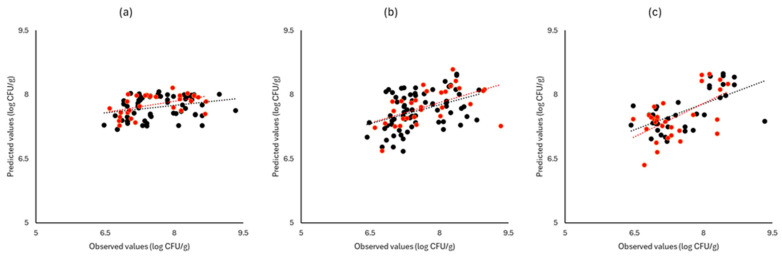
Observed versus predicted values for the estimation of TVC counts on rocket samples for FTIR (**a**), benchtop-MSI (**b**), and portable-MSI (**c**). Prediction (•), Validation (•). Dashed lines represent the linear trendline.

Figure 6, Figure 7 and Figure 8 display the observed versus the actual values of TVC for validation and prediction using PLS-R for the different sensors and examined cases. Dashed lines indicate the linear trendline (y = ax + b). For all examined cases (white cabbage, romaine lettuce, and rocket), the correspondence between the actual and observed values was not satisfactory.

As demonstrated in Figure 6, Figure 7 and Figure 8, deviations from the ideal y = x line can be observed for all instruments and samples used in both prediction and validation, resulting in inadequate models for the prediction of TVC.

## 4. Discussion

In recent years, consumers’ growing interest in healthier habits has resulted in the daily inclusion of fresh vegetables as part of a healthy diet. However, fresh vegetables have a short shelf-life, and the degradation of their quality is a multiparametric process, mainly due to the respiratory activity during their post-harvest packaging and the growth of their indigenous microbiota factors leading to spoilage [37]. The growth of their indigenous microbiota at population levels of approximately 8.0 log CFU/g and especially the growth of the specific spoilage organism (SSO) will be responsible for the end of their shelf-life [13]. Xylia et al. [13], who studied the microbial variation of fresh RTE salads during the whole year, observed that the spoilage bacteria, i.e., lactic acid bacteria and *Pseudomonas* spp., were detected during winter and spring in higher population levels (ca. 6.5 log CFU/g for LAB and up to 8 log CFU/g for *Pseudomonas* spp.) in contrast to autumn and summer, where lower population levels (1 log lower) were recorded; however, they noticed that excessive handling increases microbial load and plant stress. With regards to packaging technology, modified atmosphere packaging (MAP) is commonly used to extend the shelf-life of such perishable products, mainly by delaying the growth of the SSO [38]. The respiration rate of packaged vegetables can also be controlled by MAP due to the infused gases in the packaging, which can minimize O_2_ and increase CO_2_, thereby slowing the product’s spoilage while also limiting microbial growth [39,40]. According to the literature, the recommended O_2_ concentrations in MAP during fresh vegetable storage should range between 1 and 10% [38,41], while CO_2_ concentrations should be at least 10% [42], thus leading to the reduction of aerobic bacteria growth rate and thereby extending the shelf-life of fresh salads [43,44,45,46]. The initial gas composition in the package changes during storage until equilibrium is reached around the product [47], in the case of MAP, conceptually referred to as “modified equilibrium atmosphere” [48,49]. In the present study, MAP, 10% CO_2_/10%O_2_/80%N_2_, was used to meet the above conditions for extending the shelf-life of vegetables due to packaging conditions. In the research of Hyun and Lee [50], the effect of MAP (100% CO_2_ gas) as a preservation method for extending the shelf-life of iceberg lettuce was studied. It was evident from their results that MAP resulted in lower populations of total mesophilic bacteria, *Escherichia coli*/coliform, and yeast/mold in comparison to the air packaging during the storage of the fresh vegetables; however, their visual quality was degraded since higher levels of electrolyte leakage were observed in MAP packaging rather than air-stored samples. As regards pathogen populations, Oliveira et al. [51] used three films with different permeability in O_2_ and CO_2_ to study their effect in the growth of three major foodborne pathogens (*Salmonella*, *E. coli*, *L. monocytogenes*) after being inoculated in fresh-cut lettuce. Their results showed that no significant effect was found on the survival and growth of the pathogens in relation to the composition of the storage atmosphere within the packaging of the product during storage at refrigeration temperatures. This outcome could be interpreted as an indication of the necessity of active processes (i.e., biopreservation) to mitigate the vegetables’ possible contamination.

In addition to packaging, another crucial factor is the storage at low temperatures for the reduction of the high respiratory metabolism of fresh vegetables [52]. In the present study, two storage temperatures of the salads were used, 4 and 10 °C, the first simulating the temperature of the proper operation of the home refrigerator and the second one of the refrigerators at the sale points of such products. The results showed, as expected, that at 10 °C, the microbial growth was faster than at 4 °C but without differentiating the microbial composition as well as the maximum population of spoilage microorganisms and each pathogen tested. Similar results were observed in other studies, too [41,53].

Spoilage bacteria, yeasts, and fungi are the main microorganisms found in fruits and vegetables; however, pathogenic bacteria can also be present due to the possible contamination through the food supply chain (from cultivation to consumption) [54]. Major foodborne pathogens that have been linked with fresh vegetable produce include *Escherichia coli*, *Salmonella* spp., *L. monocytogenes*, *B. cereus*, *Campylobacter* spp., *Yersinia enterocolitica, Staphylococcus aureus*, and *Clostridium botulinum* [12,54,55,56]. An infection with these pathogens can lead to mild clinical symptoms or more complex diseases [57,58].

Recent outbreaks of gastroenteritis involved Shiga-toxigenic *E. coli* after vegetable consumption [59,60,61,62]. Several studies report that *E. coli* O157:H7 is located in plant tissues, mainly from soil contamination [9,63,64]. Additionally, Wright et al. [65] studied the colonization of *E. coli* O157:H7 in the apoplastic space of root tissues and inside root cells that possibly triggers plant and cellular responses to enteric pathogen invasions. Cattle have been several times the source of enteric pathogenic bacteria, as the widespread application of compost types from animal wastes provides a route to the crop rhizosphere from which internalization into plant tissues could occur [66,67].

Postharvest practices also constitute potential pools of human pathogens that may contaminate fresh leafy vegetables, leading to a potential food poisoning or infection risk. The post-harvest handling of leafy vegetables (i.e., rocket, lettuce, cabbage, etc.), preparing them as ready-to-eat food, includes processes like washing, slicing, shredding, peeling, and packaging aimed at reducing their initial microbiological load [68]. However, together with mishandling and surface damage, these processes can be a potential source of contamination with foodborne pathogens that can be present in the processing environment [69], such as *L. monocytogenes*. The latter is an intracellular pathogen that enters humans through contaminated food. It is well known in the literature to infect fresh leafy vegetables, such as lettuce, rocket, and cabbage [54,55]. Other studies related to the existence of *L. monocytogenes* in vegetables showed that this bacterium can be everywhere in the environment, is resistant to stresses, and is very difficult to eliminate [70,71]. In addition, fresh leafy vegetables are further processed on a large scale before consumption through various products, such as sandwiches and RTE salads.

A matter of great importance is the estimation of the expiration date of RTE salads. RTE salads are minimally processed vegetables, and by reaching their expiration date, they begin to show defects such as wilting, browning (the loss of green color), and the appearance of musty odors and unpleasant flavors that result in reduced acceptability by consumers [72]. Thus, in addition to microbial spoilage, fresh leafy vegetables may be organoleptically rejected due to enzymatic browning [73]. In the present study, the organoleptic rejection from the respective sensory panel occurred due to the appearance of browning on the leaves, even though the microorganisms were at the same population levels between the cases for all salads tested. This is evident in Figure 1, where the control case is rejected because of the leaf browning. Each plant species reacts in different ways in the browning of leaves through storage. The rocket salad is one of the most studied species in this respect, as it shows great sensitivity [41,53]. Lettuce is sensitive, too [74]. On the other hand, the cabbage is the least sensitive, but the organoleptic rejection of this vegetable is evident through browning, too. Altered phenol metabolism is believed to be involved in the leaf browning of vegetables [74]. The first step in phenol metabolism is the conversion of the amino acid l-phenylalanine to trans-cinnamic acid by the enzyme phenylalanine ammonia lyase (PAL). Some researchers suggest PAL as an indicator of shelf-life in some fresh vegetables [75,76,77].

Protective LAB cultures have been previously used to extend the shelf-life or increase the safety of fresh and minimally processed vegetables [16,17,22,23]. The preservation abilities of LAB can be attributed to the production of antimicrobial compounds (i.e., organic acids, hydrogen peroxide, and bacteriocins) [24,25]. Bacteriocins, which are natural antimicrobial peptides and are considered safe natural biopreservatives, can kill or inhibit bacteria growth [24,26,27]. Currently, the use of these molecules in solutions during washing procedures for minimally processed vegetables is being studied [18]. Additionally, several authors reported a 1.2 to 2.7 log unit reduction in *L. monocytogenes* loads in vegetables that have been washed with solutions containing bacteriocin [16,17,25,78,79]. The *L. pentosus* FMCC-B281 strain used in the current study was selected based on previous research that showed (a) its high survival rate during the storage of green table olives in MAP [30], (b) better organoleptic properties in comparison with the control [30], (c) probiotic properties in vitro [28], and (d) higher adhesion rates to Caco-2 colon cancer cells compared to a probiotic reference strain [29]. In the current work, *L. pentosus* B281 was used as a well-established LAB that can inhibit pathogens such as *L. monocytogenes* and *E. coli* in fermented vegetables [28]. In contrast to the former results [28], the addition of this LAB strain and its supernatant had no effect on microbial inhibition (pathogens or spoilage bacteria populations); however, it delayed organoleptic rejection due to the better appearance of the product. A possible explanation is due to the acids produced in the supernatant (S samples) or from the microorganism (F samples) in the product, which help the latter retain its organoleptic characteristics. In this respect, similar results with the current study were obtained in the study by Poimenidou et al. [80], where the tested washing treatments, which contained acids (i.e., vinegar, lactic acid, etc.), had a positive effect on spinach and lettuce color. However, the application of LAB strains with bioprotective properties may attribute an acceptable appearance to fresh-cut vegetables and elongate the shelf-life, thus mitigating food waste.

Due to the rapid rate of deterioration of fresh vegetables and to quantify the effect of all factors contributing to the growth of microorganisms, the use of rapid, non-invasive spectroscopy-based methods to assess the microbiological quality of such products is of great importance [53]. These methods, in tandem with machine learning approaches (algorithms widely used in the literature) and predictive modelling, may offer significant decision-support tools to the vegetable industry, capable of rapidly estimating the remaining shelf-life of fresh-cut salads [41,53]. In this context, the objective of the present study was the comparative assessment of different non-invasive sensors and machine learning approaches for evaluating the microbiological spoilage of ready-to-eat leafy vegetables. According to the findings of this study, following well-established procedures, the sensors did not lead to satisfactory results regarding predictions, suggesting that there is not a single combination of analytical approaches or algorithms that could be applied successfully to all food products and throughout the food supply chain. In the current study, a possible explanation could be that among the different treatments, the same population levels of microorganisms but with different organoleptic evaluations were observed. In the future, a different approach of analysis using different analytical techniques or other machine learning algorithms in tandem with the analysis of a higher sample number, taking into account, e.g., the initial status in terms of freshness or production seasons without considering the microbial loads, will maybe lead to better results.

## 5. Conclusions

In conclusion, the applied bioprotective strain can extend the shelf-life of the RTE salads without having a significant effect on the growth of the two examined pathogens. Indeed, the pathogens increased for romaine lettuce and rocket independent of the treatment. According to a sensory evaluation, organoleptic rejection was faster in the control samples in contrast to the S and F samples, where their shelf-life was elongated by 1 or 2 days. FTIR and MSI analyses did not lead to satisfactory results for predicting the actual microbiological quality of salads. Future studies investigating the use of other bioprotective cultures or exploring the interactions between the bioprotective culture(s) and the indigenous microbiota and evaluating their impact on the physiology of pathogens and their potential virulence should be designed and implemented. Finally, the development of more robust predictive models for the shelf-life estimation of leafy vegetables is of high importance. In that sense, the application of various machine learning approaches is required alongside the increase of the sample size, covering real-life scenarios (different producers, origin, storage conditions, or seasonality) in leafy vegetables safety and spoilage.

## Figures and Tables

**Table 1:** The performance indices of the PLS-R models for the TVC evaluation of rocket, cabbage, and romaine lettuce samples using different sensors.

Sample	Instrument	Selected Region (cm^−1^)		RMSE	R^2^	Slope	Offset
Rocket	FTIR	870–1800	Training	0.667	0.132	0.132	6.698
			Validation	0.157	0.600	0.176	6.438
			Prediction	0.614	0.041	0.114	6.836
	Benchtop-MSI	-	Training	0.549	0.402	0.402	4.612
			Validation	0.585	0.288	0.319	5.261
			Prediction	0.572	0.049	0.291	5.431
	Portable-MSI	-	Training	0.513	0.454	0.454	4.121
			Validation	0.512	0.222	0.527	3.577
			Prediction	0.555	0.387	0.404	4.556
White cabbage	FTIR	870–1800	Training	1.145	0.279	0.279	4.258
			Validation	1.244	−0.078	0.232	4.518
			Prediction	1.464	0.274	0.237	4.515
	Benchtop-MSI	-	Training	0.950	0.466	0.466	3.058
			Validation	1.212	0.250	0.636	2.109
			Prediction	1.244	0.178	0.379	3.344
	Portable-MSI	-	Training	0.559	0.814	0.814	1.227
			Validation	0.633	0.787	0.751	1.731
			Prediction	0.722	0.756	0.699	1.817
Romaine lettuce	FTIR	870–1800	Training	0.737	0.396	0.362	4.905
			Validation	0.865	0.226	2.444	5.902
			Prediction	0.847	0.125	0.319	5.318
	Benchtop-MSI	-	Training	0.702	0.466	0.466	4.082
			Validation	0.429	0.721	0.306	5.264
			Prediction	0.708	0.331	0.310	4.564
	Portable-MSI	-	Training	0.495	0.649	0.649	2.210
			Validation	0.583	0.287	0.514	3.641
			Prediction	0.604	0.347	0.376	4.581

## Data Availability

Dataset available on request from the authors.
